# Supplementary material for: Exploitation and exploration in text evolution. Quantifying planning and translation flows during writing
Source: PLoS One. 2023 Mar 30;18(3):e0283628. doi: 10.1371/journal.pone.0283628 (PMC10062643; doi:10.1371/journal.pone.0283628)
Supplement: S1 File — (PDF) [file pone.0283628.s001.pdf]

Supporting Information: Exploitation and exploration in text evolution. Quantifying planning and translation flows during writing.

D. Ruggiero Lo Sardo[1,2,3,*], Pietro Gravino[4,1,2], Christine Cuskley[5], Vittorio Loreto[4,1,2,6],

1 Sony Computer Science Laboratories Rome, Joint Initiative CREF-SONY, Centro Ricerche Enrico Fermi, Via Panisperna 89/A, 00184, Rome, Italy
2 Centro Ricerche Enrico Fermi, Via Panisperna 89/A, 00184, Rome, Italy
3 Complexity Science Hub Vienna, A-1080 Vienna, Austria
4 Sony Computer Science Laboratories Paris, 6, Rue Amyot, 75005, Paris, France
5 Language Evolution, Acquisition and Development Group, Newcastle University, Newcastle Upon Tyne, NE1 7RU, United Kingdom
6 Physics Department, Sapienza University of Rome, Piazzale Aldo Moro 2, 00185 Rome, Italy

* donaldruggiero.losardo@sony.com

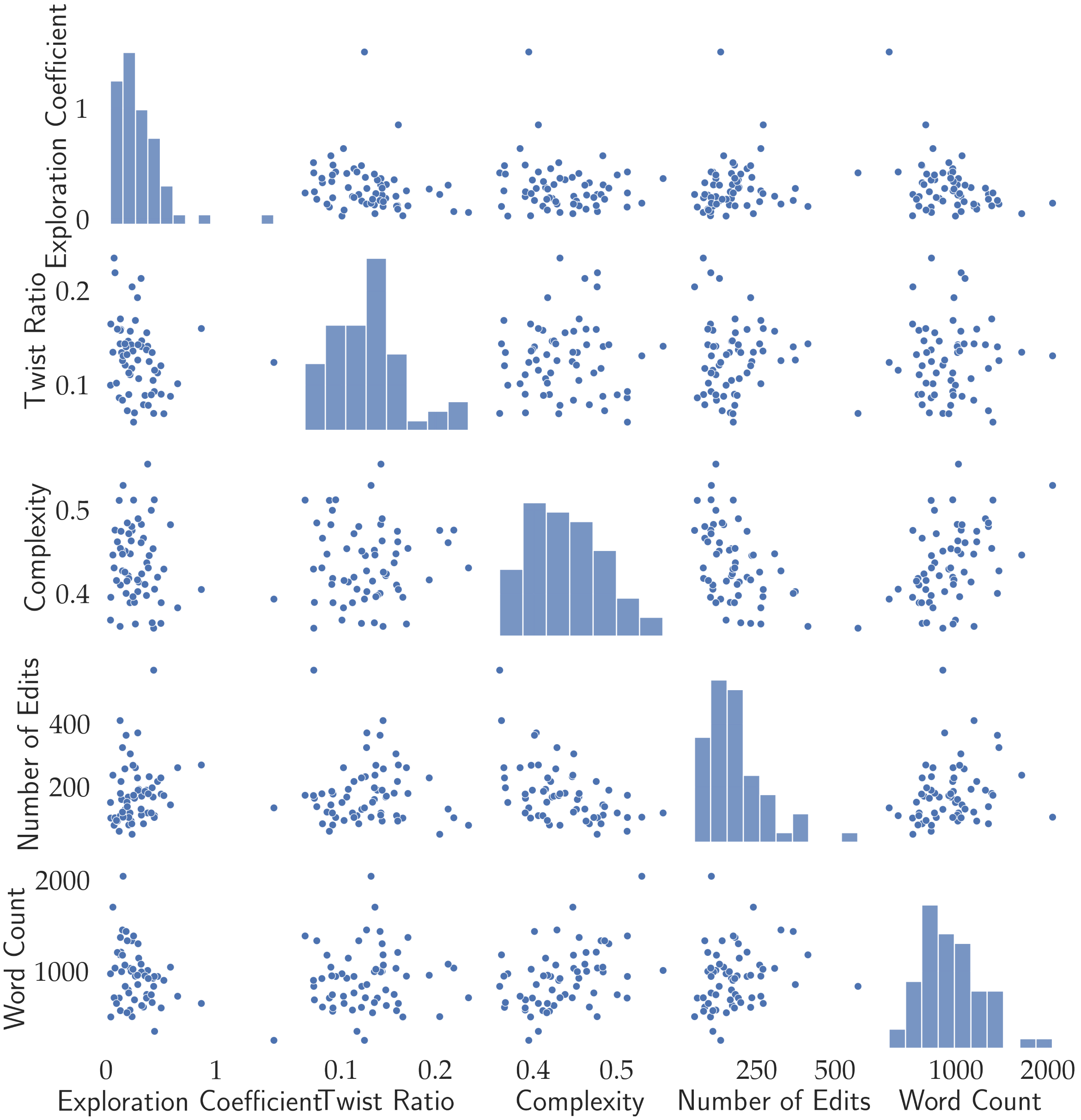

Figure 1: A matrix of scatterplots displaying the relations between metrics on the drafts of SEW participants. The diagonal elements display the distribution of the metric.
