# Supplementary material for: Exploitation and exploration in text evolution. Quantifying planning and translation flows during writing
Source: PLoS One. 2023 Mar 30;18(3):e0283628. doi: 10.1371/journal.pone.0283628 (PMC10062643; doi:10.1371/journal.pone.0283628)
Supplement: Attachment — Submitted filename: Rebuttal.pdf [file pone.0283628.s002.pdf]

Rebuttal letter for “How do authors navigate the complexity of the writing process?”

We are thankful to the Editor for providing constructive feedback, and for her interest in allowing this submission to continue. We are also grateful to the Reviewers for providing insightful and detailed feedback that has helped to significantly improve the clarity and depth of our study. Our replies are provided through indented text beneath each comment from the Editor and Reviewers. When our replies incorporate the direct revision from the manuscript in quotations, we further indent these quotations within our reply.

Once again, we are grateful for the opportunity to submit our revised manuscript to PLOS ONE, and we are happy to readily implement any additional improvements that arise in the review of our updated submission.

Response to the Editor

> We are very thankful to the editor for considering our work and for the possibility to address the points raised during the reviewing process.

However, they both found some critical issues that prevented me to consider this version of the manuscript ready for publication. I agree with them that the measures you introduced need to be better described and discussed, also with respect to other measures present in the literature; in addition, the study needs to be more appropriately situated at the theoretical level.

> We thank the editor for their interest in our work. Regarding the contextualization of the metrics, their description and discussion we have edited the introduction adding more bibliographical references, and a paragraph aimed at better clarifying the scope of these measures. Following reviewer-2’s comment, we have moved part of the results, dedicated to the introduction of the metrics to the general public, to the methods section. We further address the reviewer’s comments in detail in the following sections of this document.

Finally, but very importantly, I recommend to make data on which analyses are based available, clarify what type of consent has been asked to participants and whether the study has been approved by an ethical Committee.

We decided to follow your recommendation and to make the data on which our analyses have been performed available. They can be found here: https://github.com/SonyCSLParis/writingcomplexity.
During the registration, participants were informed about the data gathering and about the aims of the experiment and they accepted to:

- release the rights on the whole production at the workshop to Sony CSL, Sapienza and Inserm for scientific purposes only (they keep the intellectual property rights on my production);

- allow the use of my personal data for scientific purposes only, provided that they are not disseminated other than in aggregate form.

Finally, all the experiments were conducted in accordance with the organisers ethical guidelines (SONY, Sapienza University, and Institut National de la Santé et de la Recherche Médicale, i.e. INSERM of France). Furthermore, the procedures were validated by the SONY Computer Science Lab internal management board. The experiments were also reviewed and approved by each hosting institution: Complexity Science Hub Vienna (Prof. Stefan Thurner), The Albert Einstein Center for Fundamental Physics in Bern (Prof. Gilberto Colangelo), the Institut des Systemes Complexes in Paris (Prof. David Chavalarias).

Response to Reviewers

We wish to thank the attentive work of the reviewers. We strongly believe that thanks to their suggestions and indications our work is much clearer and better situated in the wider conversation on the writing process. We took their advice and insight to heart and propose a series of changes that we describe in detail in the following.

Reviewer #1:

1. Theorising in the introduction is imprecise and insufficiently rooted in existing theory and research. There is too much grand gesture here, I think, and too little incremental science. I hope the following two examples illustrate what I mean. "There is ample evidence that the writing process influences the quality of the final text and that mastering self-regulating strategies leads to higher coherence and, in general, better texts [3–12]. " The first part of this claim is trivially true. Text only results from the processes used to create it. There is not other way in which it appears on the page. The authors might here be thinking just about the particular strategic activity that a writer engages in - the ways in which they choose to regulate their own behaviour as they produce text affects text quality independently of their content knowledge and linguistic skills. If so, that's what they need to say. However, that's an empirical claim, and there's counter evidence. The list of papers referenced here contains a whole range of research, including papers on the effects of first grade instruction - a literature which does not come down strongly on the side of students needing explicit writing

strategies (I would and have argued). It also includes one of my papers where our main finding is actually no relationship between process and text quality.

We thank the insightful advice of the reviewer and have changed the text accordingly:

Lines 12-15:

> *“The evidence on whether particular sequencing strategies, explicit or instinctive as they may be, contribute to the quality of the final text and whether mastering self-regulating strategies leads to higher coherence and, in general, better texts is the subject of ongoing research [3– 12]”*

"As such, many have argued that writing is a complex task. The writer has a mental model of her/his work that must adapt since the whole is affected by variations of the constituent parts, such as individual sentences or even words [13]. We postulate that these adaptations trigger shifts from one writing phase to another. During these shifts in the cognitive task, the model of the text can undergo creative reconstruction. The identification and analysis of these events, as well as the study of other patterns of the process, can be crucial to improving writing strategies." Several claims here that need to be properly worked through and situations in existing research and theory. (1) "The writer has a mental model of their work." I am not sure what is meant by "work" here. This could be one or both a representation of the macrostructure (or surface form?) of the text produced so far, or a message-level representation of what they aim to communicate, or what what they believe that they have communicated so far. There are other possibilities. It's also not clear that these representations actually exist or play an important role in written production. They may do, but the evidence isn't there, to my knowledge. See, for example, https://doi.org/10.1017/ - and I have argued that similar processes are likely to occur in writing. It is possible that the macrostructure that makes text cohere into a message is emergent rather than something that maps directly onto any mental model, even one that has changed during writing. (2) "that must adapt since the whole is affected by variations of the whole is affected by individual sentences…or even words" Again, I'm not sure what this means. Words and sentences do not just appear. They are output from the writer's mental representation (at whatever level, assuming this exists) and so should necessarily cohere with existing representations. If the writer was a reader then this claim would make perfect sense. There may be a sense in which it is true for writers, but this is a theoretical claim. If it's important, then it needs unpacking and supporting. If it's not then if should be dropped. (3) " We postulate that these adaptations trigger shifts from one writing phase to another. During these shifts in the cognitive task, the model of the text can undergo creative reconstruction." I'm not sure what the authors are thinking of here. Something like you output a particular sentence that has a meaning that surprises you (for some reason). This then means that you decide to revise your text in light of new ideas? Are there other possibilities? If not, then just say this. If there are, then elaborate. Then, why does the shift in the task result in reconstruction (I'm assuming of mental representation of message? Isn't it the other way round - you change your ideas and so see the need to revise your text. And so on. My point here is definitely not that the authors should present a full blown theory of mental processing underlying text production, or that this is necessary, or that the authors intended meaning is wrong. They do, however, need to avoid vague hand-waving. If they are going to make a priori claims about mental processing underlying text production these need to be explained

clearly and precisely - avoiding imprecise use of jargon - rooted in existing theory and research, and as far as possible evidenced.

We are thankful to the reviewer for indicating the lack of clarity in this passage of our work. We are hopeful that the revised version will clarify the claims of our work.

Lines 17-26

> *"The writer has a rough idea, a model, of what her/his final output will be [13]. This model must adapt since the imagined final version must conform to variations of its constituent parts, such as individual sentences or even words [14, 15]. Examples might be rhetorical solutions that do not satisfy the author or instances where through the writing process some unforeseen clarity on the content or new idea is found by the author himself. We postulate that these adaptations trigger shifts from one writing phase to another, such as from sentence generation to revising or even planning. The identification and analysis of these events, as well as the study of other patterns of the process, can be crucial to improving writing strategies."*

2. The authors should carefully situate the new methods that they described in previous efforts to extract similar information from writers text-generation processes. "Here for the first time" in the following - "Here, for the first time, we uncover those processes by recording, through a suitably dedicated software, all edits and actions in the making of a text, from the early attempts to the finishing touches." - is not justified, I think (or at minimum the authors need to strongly evidence this claim). To be clear, I think the methods that they describe are of potential value, and I would be excited to see them reported in a general-interest journal. However, they do not represent that paradigm shift that the authors are claiming. In particular, they are not an alternative to approaches involving think aloud (although it's a long time since I've seen papers using these methods), experimental manipulations, and keystroke logging. These are currently represented as alternative approaches to a single research goal. In fact, they serve quite different purposes, and are not in any sense mutually exclusive. This is not an appropriate framing for their new methods. What the authors want to demonstrate is how texts evolve over time, particularly in contexts (relatively rare but important) where the text is subject to extensive revision. This is an absolutely worthwhile goal. It is, though, something that has been addressed before. I've not used these methods in my own research, but if I was going to I would search forward from Eklundh, K. S., & Kollberg, P. (2003). Emerging discourse structure: computer-assisted episode analysis as a window to global revision in university students' writing. Journal of Pragmatics, 35(6), 869–891), which is implemented in Inputlog (cited in the current paper), Also graph-based methods (see https://doi.org/10.1075/z.194.; https://www.ggxlog.net/). Another example is the choice of revision unit problem that they authors address from lines 133 onwards. Their solution sounds good, but the problem has been addressed in previous research (I think) - see papers here https://research.tue.nl/en/. I think a review of this literature needs to be the main focus of the authors' introduction. I am definitely not claiming that the authors' cover the same ground as these, but their focus needs to be on how the methods the they describe move beyond these.

We are thankful to the reviewer for helping us no, better situate our research in the context of revision and editing strategies. We believe our research provides an improvement upon some of the cited methods since it requires only versioning data and because in comparison to other graph techniques, the definition of a node is both flexible, and also grounded in the construct choice. We are hopeful that the changes made to the text will help better contextualise our work and also clarify elements of novelty in our research.

Author summary:

> *"Here we uncover those processes by recording, through a suitably dedicated software, all edits and actions in the making of a text, from the early attempts to the finishing touches."*

Lines 39-41, 62-64, 92-96.

> *"Existent work has used three main experimental setups that give access to different kinds of information on the writing process:[...]"*

> *"Here, we propose a new method that presents a much smaller overhead than previous work such as S-notation [15], since it does not require keystroke logging to reconstruct the activity of the author."*

> *"In the subsection Exposing Complexity, we introduce a representation of the writing process that details where and when the author has made changes to her/his draft, and improves upon previous attempts by proposing a way to change scale from characters to words, sentences, paragraphs and other segmentations of text [28]"*

3. The measures that the authors report are interesting and potentially valuable. On the basis of what they report I do not get a strong feel for construct validity. This is for two reasons. First, the authors are vague about the construct that they aim to measure. It feels at times that the measure is driving the construct and not the other way round. For example I am not clear what it means for a writing process to be "structured" as in "The Complexity Score aims to quantify how structured the writing process is." (line 134). The authors illustrate this with "only one sentence was edited repeatedly, the process is highly organized. If all sentences were edited a comparable number of times, the process is indistinguishable from a random one" (line 162). I can see how these two cases are different, and that much more revision occurs in the latter case, but I'm not sure why the second one is less structured or organised. The second reason is that, even as a reader who is used to abstract representations of texts and writing processes, I find it very difficult to relate indices and plots to my understanding of how texts might change or how writers might engage in cycles of revision. I don't feel that I have a strong enough understanding of the measures that the

authors propose to make recommendation for how this gap might be bridged, but something is needed. Again, to be clear, I am not at all criticizing the authors for seeking to abstract features of the ways that a writer or writers interact with and develops their texts and specific indices. I just need a stronger feel for what is being measured.

We are thankful to the reviewer for highlighting the need for greater clarity both on the aims of our measures and their meaning. We hope the changes we made to the text will help the reader in these regards.

Lines 90-99, 190-199:

> *"The outline of the paper is as follows. In materials ad methods we discuss the data gathering procedures and the three original metrics introduced in this work and discussed in the results and discussion. In the subsection Exposing Complexity, we introduce a representation of the writing process that details where and when the author has made changes to her/his draft, and improves upon previous attempts by proposing a way to change scale from characters to words, sentences, paragraphs and other segmentations of text. We use this representation to investigate the distribution of work along the length of the draft and propose a measure of complexity that differentiates the space between randomness and ``machine-like'' writing, i.e., writing the final version directly without any revisions."*
>
> *"To clarify, when we talk of structure in the context of complexity, we talk of the structure of edits as mapped onto the unidimensional space of the text, i.e. the x-axis of Fig. 1 (b). If the author edits specific elements more than others, the editing process structure is simply organized around a few focal points. If the edits are more evenly distributed across the space of the text, they are the result of a more complex and less predictable process. It is important to remark that we are not looking at the dynamical process of writing, that happens in the space of the text and in time. We are focusing on the organization of the author's attention across the text. The distribution of edits in the text space represents the distribution of the author's effort. We use the effort as a proxy for the author's attention."*

Reviewer #2:

[...]

Abstract

In the final sentence, I suggest substituting the word "learning" because the study does not treat learning in the writing process.

We thank the insightful comment made by the reviewer and have updated the abstract accordingly:

> *"Our results and the new quantitative tools deployed have the potential to foster the discussion about the nature of writing and give the processes an evolutionary perspective, aiming at more creative and impactful writing processes."*

Introduction

[...] My impression is that references are scarce. For example, in line 26 there is a list of fields that studied text analysis but only one reference. The following paragraph on cognitive sciences presents only one reference for each method, even if the scientific works are many. Here, I would use a different term instead of "pedagogy" (line 34). Studies are generally concentrated on learning progress during the education path or in analyzing the writing level in a specific grade. "Pedagogy" is more related to "educational facts", while most of the cited studies are in the field of developmental and educational psychology or general psychology and linguistics. In the same paragraph (lines 47-48), it is not so clear to me why the authors compare keystroke logging to technics such as functional magnetic resonance, GRS, EEG. These technics are common to cognitive psychology but are quite different from each other. I would omit that sentence.

We are thankful to the reviewer for indicating the lack of clarity in these passages of the introduction. We hope the updated text will provide the needed clarification.

Lines 51-55

> *"Lastly, keystroke logging uses software that records each keystroke to characterize the evolution of the draft. Keystroke logging is an unobtrusive technique often used in conjunction with other observation techniques such as eye-tracking, functional magnetic resonance imaging (fMRI), galvanic skin response (GSR) and electroencephalography (EEG), to uncover relations between the writing process and the author."*

The last paragraphs of the introduction present the study and the outline of the following sections.

I would suggest focusing more clearly on the aims of the work (analyzing complexity, exploration, and exploitation in text generation) and briefly introducing the three original metrics developed.

It seems that the introduction lacks presenting the intents of the study or, in other words, why those metrics are chosen to analyze the writing process. A more detailed explanation of how each metric is devoted to analyzing a specific behavior would make it easier to read the methodological section. Only in Results the reader has a clearer idea.

We thank the reviewer for the suggestion and have edited the paper to better clarify the link between the chosen metrics, their explanation and the goal of each metric.

We have included a paragraph in the introduction to clarify the goals of the study and how the metrics contribute to that aim. The sections on each metric we have moved from the Results section to the Methods section in order to keep the structure of our work more streamlined. We are hopeful that with this change the reader is better equipped to understand the Methods section.

Finally, in line 62 "we also present a new dataset composed of …". I suggest rephrasing given that the authors are not presenting a dataset. As I understand, the data will be not public for privacy reasons. This poses some doubts about the absence of an ethical statement and the collection of personal information.

We are thankful to the reviewer for the comment. Regarding the dataset, we finally decided to provide anonymized information on the author in the form of edit sequences (as described in the method section), number of versions, the workshop label and the edit distance between all versions at this address:

https://github.com/SonyCSLParis/writingcomplexity

Regarding the consent, we have provided informed consent forms to each author for the scientific use of their data (see response to the editor).

Experimental Setup. Table 1 presents the number of participants in the workshop. However, the authors declare that "the analysis includes only data for participants who's (whose?) draft change at least 10 times". How many participants were discarded? Please, report the final sample size.

We are thankful to the reviewer for pointing out this lack of clarity. The numbers in the table refer to active participants, so participants that authored at least 10 versions of their text. We have updated the text to make this point clearer.

Lines 117-120:

> *"There were no exclusion criteria for the participants but the analysis includes only data for participants whose draft changed at least 10 times, to control for active participation (numbers in table 1 refer to active participants)."*

Measuring Complexity. Line 146, I suggest introducing the scales you tested. The reader understands only later that are characters, words, and sentences.

We thank the reviewer for the suggestion. We are hopeful that the edited text states more clearly the scales we have tested.

Measuring Exploration. Please, define "shortest sequences" and shortest path".

We are thankful to the reviewer for pointing out the lack of clarity in this passage. We hope the changes to the text address these issues.

Lines 218-228:[1]

[1] Text delimited by the "$" is to be interpreted as mathematical formulae in latex notation.

“Knowing the final version of the draft, we can imagine many different processes that led to that version, each of which can be represented as a sequence of edits. [...] The set of points such that $d_{0f} = d_{0t}+d_{tf}$ is, by definition, the one where all the shortest sequences of edits lie that lead from the first version to the last. If, at a given time t, the author is not on one of the shortest sequences, then she/he has been, to some degree, exploring the conceptual space of her/his work. Let us define as $H(t) = (d_{0t}+d_{tf} - d_{0f}) / d_{0f}$ the distance from one of the shortest sequences, normalized by the distance between first and last version (in order to compare different texts)”

The last sentence of the subsection is not clear and poses doubts on the choice to use characters and not sentences as for the complexity measure. Please define "the structure of individual change".

We thank the reviewer for indicating the lack of clarity and hope that the updated text better explains our meaning.

Lines 232-235:

“For this analysis, contrarily to the previous one, we will treat each character separately when computing edit distance since we are aim to quantify the amount of effort that was devoted to this exploration, rather than the structural changes that where made to the draft.”

Exploration. Lines 282-284. Are the minor edits counted in the analysis of exploration? Could this be a limit of the metric?

We thank the reviewer for the interesting question. To clarify, minor edits are counted in the analysis of exploration. The size of the edits is the main reason their contribution is not seen as a major limitation. We envision a more precise metric that can distinguish between the two cases, that relies on edit classification that will be developed in future work.
