# Supplementary material for: Exploitation and exploration in text evolution. Quantifying planning and translation flows during writing
Source: PLoS One. 2023 Mar 30;18(3):e0283628. doi: 10.1371/journal.pone.0283628 (PMC10062643; doi:10.1371/journal.pone.0283628)
Supplement: Attachment — Submitted filename: Response to Reviewers.pdf [file pone.0283628.s003.pdf]

Rebuttal letter for “How does text evolve? Quantifying the dynamics of exploration and translation during writing”

We are thankful to the Editor for providing constructive feedback, and for her interest in allowing this submission to continue. We are also grateful to the Reviewers for providing insightful and detailed feedback that has helped to significantly improve the clarity and depth of our study. Our replies are provided through indented text beneath each comment from the Editor and Reviewers. When our replies incorporate the direct revision from the manuscript in quotations, we further indent these quotations within our reply.

Once again, we are grateful for the opportunity to submit our revised manuscript to PLOS ONE, and we are happy to readily implement any additional improvements that arise in the review of our updated submission.

Response to the Editor

We are very thankful to the editor for considering our work and for the possibility to address the points raised during the reviewing process.
In response to overall comments about clarity and framing from a cognitive science perspective, we have brought on an additional co-author with expertise in language and cognition.

Response to Reviewers

Reviewer 1
We thank the reviewer for engaging with the work once again and providing valuable feedback. Below, we briefly summarise each of their numbered comments alongside how we addressed them.

1. “*[the authors should] carefully specify the problem that needs solving, looking in some detail at existing methods, and then presenting the new method in such a way as to make clear what it is adding to what already exists*”.
    a. We have done extensive rewrites to frame our methods within the well-accepted framework of writing as a process that takes place in three interrelated phases: planning, sentence generation (translation/transcription), and revision. Specifically, we have detailed how our measures are a new way of looking at cycles of planning and translation during the revision phase in

the introduction, as well as referencing this model more concretely in explanations of our measures and results. While the interrelatedness of these phases is well accepted, we contend that they are often measured in isolation, and our measurements provide a new way to look at them in an integrated way.

2. "*[the authors] should drop all claims about underlying cognitive processes…[including] a 'complete unit of thought' …[and] 'effort as a proxy for the author's attention'...[where authors discuss these issues with respect to their own data] it needs to be strongly caveated*"
    a. We have removed reference to units of thought and attention; we agree these are either unclear or not measurable in our dataset.
    b. We have, however, kept the notion of effort: we contend that particularly our complexity measure gives a good indication of where in the text a writer focused their effort, but have added an additional explanation that this is a *spatial* measure, not a temporal one (i.e., it doesn't speak to how much time a writer spent making an edit, but where in the text they concentrated their editing efforts; lines 239-251).
    c. In terms of the sentence-level unit we use for the complexity measure, we've removed reference to "unit of thought", but instead clarified that the sentence level is where, in our dataset, we can best capture related adjacent edits as a single event, and we point out that this is consistent with previous work indicating that the translation aspect of sentence generation integrates with working memory mainly at the sentence level (Kellogg, 2004)[1]
3. "*I am struggling to understand the methods that the authors describe…I need a worked example, or a more detailed explanation to understand*"
    a. We've added detail to the explanation of our methods throughout to add clarity, particularly in terms of the framing we introduce in the introduction.
    b. We have restructured the manuscript so that the results for each measure are adjacent to the introduction of the measure itself. In addition to the detail added in (a), this makes the aims of each measure clearer with respect to our specific dataset.
    c. We have updated Figure 3 in order to better explain the geometrical elements used to compute the twist ratio with a concrete example of an author's draft trajectory

Reviewer 2

We thank the reviewer for their feedback, especially clarity about issues resolved in the first round of revision. We respond to their most salient points below

1. "*The methodological section should be devoted to understanding the technical details of the metrics, not their theoretical functions which should be better described in the introduction. I suggest rethinking how to introduce the metrics keeping in mind the large audience that the manuscript could reach*"
    a. This comment was echoed by reviewer 1 (point 1); we have provided more detailed theoretical framing throughout the introduction and situated our results and metrics within this new framing.

---

[1] Kellogg, R. T. (2004). Working memory components in written sentence generation. *The American journal of psychology*, 341-361.

2. “*text planning, editing and revising processes are previously studied. However, the literature review presented here does not say how the previous studies addressed and measured these processes.*”
    a. We have now put previous literature in a dedicated section ‘Understanding writing’. We focus on the fact that while previous studies acknowledge the interrelatedness and non-linearity of the well-known three phases of writing (planning, sentence generation, revision), they often measure these in isolation. The motivation for our measures is therefore to look at these together - particularly at revision as sub-cycles of planning and translation.
3. “*The authors re-formulate the sentence about keystroke logging and other techniques such as EEG, and fMRI…such techniques are just mentioned without any citation of the studies they refer to.*”
    a. We have now added specific supporting literature for this on lines 57-59.
4. “*It also lacks [explanation of] the contribution of these [keystroke] studies to text production analysis…Keystroke logging is not conceived to analyze text evolution as a whole or where the author focuses her/his effort.’ Is this really true? Can you justify the sentence? What do you mean by ‘text evolution as a whole’? I suppose that keystroke logging (also when powered with physiological or neural measures) is devoted to understanding deeply the cognitive effort of the writer. Knowing the studies that the authors are referring to would be helpful to disentangle these doubts*”
    a. On lines 55-72, we clarify the focus of previous keystroke logging studies, which differs generally in its aims from our approach (which does not use keystroke logging in any event). We point out that it often isn’t used in isolation, but alongside other methods, and nonetheless is used to look at when writers focus their efforts in terms of the timecourse of their writing, and not where in the text they are focused (lines 69-72). We also return to this issue in the discussion, where we point out that like keystroke logging, our measures relying on versioning (which has lower overhead than keystroke logging) could be usefully combined with more traditional approaches like protocol analysis and task-driven experiments (lines 469-474).
5. “ *I think that the authors should reformulate the expression ‘writing cloud’ as they have not mentioned what is a writing cloud yet. Alternatively, they should define it.*”
    a. We have made changes to the abstract to indicate a clear definition of what we mean by “writing cloud”, as well as flagging for the reader that this is a concept we introduce in the course of the work. Within the Complexity results section (lines 260-300), we have also added additional exposition as to what this measure shows, and what we might expect it to look like under different conditions (e.g., where the writer focused edits more narrowly).
