# Supplementary material for: Exploitation and exploration in text evolution. Quantifying planning and translation flows during writing
Source: PLoS One. 2023 Mar 30;18(3):e0283628. doi: 10.1371/journal.pone.0283628 (PMC10062643; doi:10.1371/journal.pone.0283628)
Supplement: Attachment — Submitted filename: Rebuttal letter for How does text evolve MK2.pdf [file pone.0283628.s004.pdf]

Rebuttal letter for “How does text evolve? Quantifying the dynamics of exploration and translation during writing”

We are thankful to the Editor for providing constructive feedback, and for her interest in allowing this submission to continue. We are also grateful to the Reviewers for providing insightful and detailed feedback that has helped to significantly improve the clarity and depth of our study. Our replies are provided through indented text beneath each comment from the Editor and Reviewers (in green). When our replies incorporate the direct revision from the manuscript in quotations, we further indent these quotations within our reply.

Once again, we are grateful for the opportunity to submit our revised manuscript to PLOS ONE, and we are happy to readily implement any additional improvements that arise in the review of our updated submission.

Response to the Editor

We are very thankful to the editor for considering our work and for the possibility to address the points raised during the reviewing process.

Response to Reviewer #1

Thank you for taking the time to review our paper and for your positive comments regarding the potential significance of our study to the academic community.
We are grateful for your congratulations on the new framing of our methods, and we are pleased to hear that you found the summary to be a competent and comprehensive description. We are glad that you appreciate how we worked the new framing into the rationale for our measures and interpretation of findings.
We thank you for the specific comments you made throughout the text. However, we would like to address your main concern about the method's clarity.
We considered separating the description and results in a previous stage of the review process but following suggestions from the other reviewer, we ended up in the current structure where each result is preceded by a descriptive introduction. We have however updated the description of our methods, thanks in no small part to your comments, and hope that this further clarification will prove to make the work easier to comprehend and more cohesive.

In the results section, in addition to what the authors already provide, I would like to see a couple of other things: Some analyses that show the relationship among measures (aggregating within writer/text). Just scatterplots with regression lines would be fine here. So are texts with a high exploration coefficient have higher or lower twist ratios, and so forth. And also relationship between aggregate measures and (a) total writing time (I realise that this is closely related to version index - and you've already done this for the exploration measure) , and (b) final text length (in words).

We thank the reviewer for the opportunity to add depth to our work. We have added a table indicating correlations among the metrics and their significance. In order to satisfy the request for scatterplots we have added a supplementary information document that contains scatters of all combinations of metrics and the aggregate measures requested by the reviewer. We are sorry we cannot really provide total writing time as such, since the workshops had diverse structures that cannot always be accounted for. We however provide plots for the relation with version counts, that as the reviewer indicates is strongly related to writing time.

Regarding your specific comments, we will make the necessary revisions to the abstract to give the exact number of junior researchers involved in the study.

- *“61 junior researchers in science wrote a scientific essay intended for a general readership.”*

We appreciate your feedback on the introduction to the paper and have revised it to accurately reflect the existing body of psycholinguistic and educational research.

- *“A body of theoretical and experimental work across psychology, linguistics, and education has developed well-accepted models of the process of writing.”*

"Here, we propose a new method that can efficiently detect patterns indicative of writing sub-cycles in much smaller datasets than existing NLP methods, presenting a much smaller overhead than previous work such as S-notation [15] given that it does not require keystroke logging to reconstruct the activity of the author." I'm not clear here what overheads the authors are referring to. Collecting keystrokes and then reconstructing all of the author's revisions is not computationally too complex (or at least is a problem that's been solved) and datasets are not huge. Versioning is an alternative approach, and there might be some practical advantages. However, I don't see this as a major contribution of the authors method. Also, given that any version could be reconstructed from a keystroke log, it would presumably be possible to apply them to keystroke data (after some post-processing). My point here is just that the methods that the authors describe contribute something conceptually valuable (and more important) than the fact that text-states were captured by versioning. Versioning isn't a main selling point (or perhaps not a selling point at all).

We agree that the use of versioning systems is not a major contribution of our work, we do however hold that the capacity of our methods to be applied to data that is more coarse-grained than keystroke logging, often already available through other systems that make use of versioning, is an advantage with respect to systems that rely exclusively on keystroke logging data. In this sense, we thank you for pointing out the lack of clarity in our work and have rewritten this paragraph in order to better state that the advantages of our methods mainly lie in the possibility of reusing data already available.

- *"Here, we propose a new method that can efficiently detect patterns indicative of writing sub-cycles in much smaller datasets than existing NLP methods, presenting a much smaller overhead than previous work such as S-notation given that it does not require keystroke logging to reconstruct the activity of the author. Since it relies on automatic versioning that is widespread across many text-editing tools, the measures introduced here can be used to investigate numerous text-based tasks for which data is already available, reducing the overhead in the data gathering process. This kind of data allows us to reconstruct a representation of the editing process which, in turn, allows us to measure its temporal complexity, and indicate when an author is in an exploratory mindset when they are effortlessly translating ideas onto the page, and when they are shifting rapidly between these tasks."*

There is a very short introduction to the key concepts of complexity, exploration, and the twist ratio at the very end of the introduction. I suggest another paragraph, at the start of the analysis / results section of the paper (p6) that unpacks these a bit more and provides orientation to the three sections that follow.

- *"In the following sections we will describe the analysis of this data divided in three main contributions: a measure of the complexity of the writing process in terms the degree to which it is a sequential process, a measure of the amount of exploratory work that goes into the writing of a text but not into the final version and a measure of the translation flow to exploration ratio in the writing steps."*

Complexity / Figure 1. I don't know whether this is because this text has been revised, or whether I've finally given it enough attention, but I think I understand the approach here, and what Figure 1 shows. However I'm am still puzzled by something. I was confused by how you handled inserted sentences without changing the index of all subsequent sentences (and therefore seeing them all as having been revised). But then I noticed "taking care to add place-holder elements for the sentences that will be inserted later in the draft so that unmodified sentences will correspond to the same positional index as the draft evolves". Good. But I don't understand how this could be achieved. Or how you handle sentence deletion. So I'm puzzled again about how this works.

We understand your confusion about how we handle inserted sentences without changing the index of all subsequent sentences. We hope we have clarified this point by expanding on how we treat deletion. In essence, we handle deleted sentences as empty placeholders so as to keep the alignment of the rest of the work.

- *"For each subsequent version of a draft after the first, we can define the edit sequence B(t). In order to correctly compare different versions we add place-holder elements (empty strings) for the sentences that will be inserted later in the draft. At the same time we keep deleted sentences as empty place holder elements. This way, unmodified sentences will correspond to the same positional index as the draft evolves."*

Twist Ratio. The explanatory panel in Figure 3, combined with the text, gives me a good understanding, I think, of how you are measuring translation flow, and I think this is a valuable idea. Beyond that I get very lost, however, in particular I don't understand lines 402 to 416. I understand twist ratio measures as angle. But I don't understand "we must decide how many dimensions to use" - dimensions in what space? I'm clearly missing something here, which suggests the need for some more careful unpacking and explanation.

For the Twist Ratio section, we have revised the text to explain the selection of the number of dimensions of the embedding space.

- *"This procedure is possible since edit distance is a well-defined metric. With the set of pairwise distances we can embed the set of versions in an euclidean space. We must, however, determine the number of dimensions to use. High dimensional spaces will guarantee that all distances between versions can be preserved, but much of the structure is lost."*

Minor point, but this "If the writer follows this "path of least resistance" very directly, this is an indicator that their original plan for the text went largely unchanged during the process of writing." isn't necessarily the case. Students often write in a very linear way, with very little revision, but this could equally be because they are simply following a train of thought - allowing previous sentences to cue what they say next- and are not particularly motivated to then restructure what they've written. This is the sort of behaviour you would expect if, for example, you asked a student to write a 5 paragraph essay on a familiar topic, in a low-stakes context (or when writing in an exam). In fact, strongly linear writing, with little revision, is, I think) less likely when writers have a clear plan. Because in this case the text they produce interacts with (is evaluated in light of) their intention. Plans set out communicative intentions for the text but (obviously) not the surface form of the text that is to be produced to fulfill these. Working out these intentions in the final text typically involves trial and error. Hence strong plans tend to result in more revision.

We thank the reviewer for pointing out that our outlining of the implications of revisions is incomplete. We have edited this paragraph to specify that the broader point we make here is that we do not observe major revisions.

- *“If the writer follows this “path of least resistance’” very directly, this is an indicator that their original plan for the text went largely unchanged during the process of writing, or that they followed a single line of thought, and in general that they did not commit to major revisions.”*

Draft vs. version: The authors use "draft", "version" and "draft version" interchangeably, I think. For me "draft " suggest a version that is identified by the author as significant (ready to show to a co-author, perhaps). Version is a better term for the text that's captured every 1 minute / 3 minutes .

We thank the reviewer for pointing out the lack of clarity on the wording in our work. We have changed all references to *versions* of a draft as such. We now use the word “draft” only to signify the final version of the work to get to it (such as in the case of “evolution of the draft”).

Response to Reviewr #2

Reviewer #2: I commend the authors on the work done in revising the manuscript. I appreciated that they have addressed my major concerns and I am satisfied with the revision. The explanation of their aims and measures is much clearer. Terminology is better specified, and the flow of the manuscript is improved.
I report an oversight: the sentence in lines 85-87 is incomplete ("In analogy with the study of strategies in evolutionary game theory we also use the term exploration to refer to planning phases, where the author tests different options for the draft, an").

We thank the reviewer for their commendation and for pointing out the oversight. We have corrected it as follows

- *“In analogy with the study of strategies in evolutionary game theory we also use the term exploration to refer to planning phases, where the author tests different options for the draft.”*

We appreciate your feedback and hope that our revisions will address your concerns and make the paper clearer and more understandable. Once again, thank you for your valuable review.
